# Identification, characterization and functional analysis of gonadal long noncoding RNAs in a protogynous hermaphroditic teleost fish, the ricefield eel (*Monopterus albus*)

**DOI:** 10.1186/s12864-022-08679-2

**Published:** 2022-06-20

**Authors:** Zhi He, Lijuan Ye, Deying Yang, Zhijun Ma, Faqiang Deng, Zhide He, Jiaxiang Hu, Hongjun Chen, Li Zheng, Yong Pu, Yuanyuan Jiao, Qiqi Chen, Kuo Gao, Jinxin Xiong, Bolin Lai, Xiaobin Gu, Xiaoli Huang, Shiyong Yang, Mingwang Zhang, Taiming Yan

**Affiliations:** 1 College of Animal Science and Technology, Sichuan Agricultural University, Chengdu, 611130 Sichuan China; 2 Luzhou Municipal Bureau of Agriculture and Rural Affairs, Luzhou, 646000 Sichuan China; 3 Sichuan Water Conservancy Vocational College, Chengdu, 611231 Sichuan China; 4 College of Veterinary Medicine, Sichuan Agricultural University, Chengdu, 611130 Sichuan China

**Keywords:** RNA-seq, *Monopterus albus*, Gonadal development, lncRNA

## Abstract

**Background:**

An increasing number of long noncoding RNAs (lncRNAs) have been found to play important roles in sex differentiation and gonad development by regulating gene expression at the epigenetic, transcriptional and posttranscriptional levels. The ricefield eel, *Monopterus albus*, is a protogynous hermaphroditic fish that undergoes a sequential sex change from female to male. However, the roles of lncRNA in the sex change is unclear.

**Results:**

Herein, we performed RNA sequencing to analyse lncRNA expression patterns in five different stages of *M. albus* development to investigate the roles of lncRNAs in the sex change process. A total of 12,746 lncRNAs (1503 known lncRNAs and 11,243 new lncRNAs) and 2901 differentially expressed lncRNAs (DE-lncRNAs) were identified in the gonads. The target genes of the DE-lncRNAs included *foxo1*, *foxm1*, *smad3*, *foxr1*, *camk4*, *ar and tgfb3*, which were mainly enriched in signalling pathways related to gonadal development, such as the insulin signalling pathway, MAPK signalling pathway, and calcium signalling pathway. We selected 5 highly expressed DE-lncRNAs (*LOC109952131*, *LOC109953466*, *LOC109954337*, *LOC109954360* and *LOC109958454*) for full length amplification and expression pattern verification. They were all expressed at higher levels in ovaries and intersex gonads than in testes, and exhibited specific time-dependent expression in ovarian tissue incubated with follicle-stimulating hormone (FSH) and human chorionic gonadotropin (hCG). The results of quantitative real-time PCR (qRT-PCR) analysis and a dual-luciferase assay showed that *znf207*, as the gene targeted by *LOC109958454*, was expressed in multiple tissues and gonadal developmental stages of *M. albus*, and its expression was also inhibited by the hormones FSH and hCG.

**Conclusions:**

These results provide new insights into the role of lncRNAs in gonad development, especially regarding natural sex changes in fish, which will be useful for enhancing our understanding of sequential hermaphroditism and sex changes in the ricefield eel (*M. albus*) and other teleosts.

**Supplementary Information:**

The online version contains supplementary material available at 10.1186/s12864-022-08679-2.

## Background

Long noncoding RNAs (lncRNAs) are transcript with a total length of more than 200 nucleotides that are little or no significant protein coding capacity [1, 2]. Compared with mRNAs, lncRNAs are more abundant in the nucleus [3–5]. They can exert their biological functions by acting on protein-coding genes in *cis* and *trans* ways [6–9]. Many studies have shown that lncRNAs are involved in several biological processes, including germ cell development [10], meiosis [11], gamete formation [12, 13] and sex differentiation [14].

LncRNAs have been found to play important roles in the reproductive development of organisms. The lncRNAs *TCONS_00025195* and *TCONS_00025196* may be involved in regulating the expression of SOX9 in human testes [15]. Most of the highly expressed lncRNAs in the testes of adult mice were found to be testis specific and were expressed at the beginning of and after spermatogenesis [16]. A total of 24,601 lncRNAs were identified in ovaries of *Columba livia*, and 148 lncRNAs were significantly differentially expressed between high and low egg production performance groups, and the target gene *FOXK2* of the lncRNA *MSTRG.7894.4* is involved in the regulation of an oestrogen receptor [17]. We identified 5317 lncRNAs and 297 differentially expressed lncRNAs (DE-lncRNAs) in *Oryzias latipes* ovaries and testes, of which lncRNA *MSTRG.14827.1* may play a key role in gametogenesis [18]. In total, 28,500 lncRNAs were identified in the ovaries and testes of *Pelodiscus sinensis*, and the target genes for the 10,495 DE-lncRNAs included several genes involved in gonadal development [19]. In addition, 962 DE-lncRNAs related to sex differentiation were found to be involved in the sex reversal induced by mifepristone (RU486) in XX *Oreochromis niloticus* gonads [20].

The ricefield eel, *Monopterus albus*, is increasingly recognized as a new model species for the study of sequential hermaphroditism and sex change [21–23]. *M. albus* is a hermaphroditic fish with reproductive traits that natural sex reversal from female to male through an intersex stage during its life cycle [22, 24]. To comprehensively reveal the molecular mechanisms of the sex change process and the potential role of lncRNAs in *M. albus*, RNA sequencing (RNA-seq) of gonadal tissues representing five sexual stages (ovary stage (OV), early intersexual stage (IE), middle intersexual stage (IM), late intersexual stage (IL) and testis stage (TE)) was performed in the present study. The functional lncRNAs, target genes and their correlations associated with both sex determination and gonad development were explored. This study provides novel insights into lncRNA function and the mechanisms of sequential hermaphroditism in fish and other vertebrates.

## Results

### Identification and characterization of gonadal lncRNAs

To study the potential role of lncRNAs in fish gonads, we used *M. albus* gonads at five developmental stages (OV, IE, IM, IL, TE) for RNA-seq and cDNA libraries*.* At least 101,886,556 clean reads were obtained. The Q20 percentage > 97.51%, Q30 percentage > 93.68%, GC content between 47.63-48.71% and percentage of error < 0.01% (Additional file 1: Table S1). These values indicated that the RNA-seq data were reliable.

**Fig. 1:**
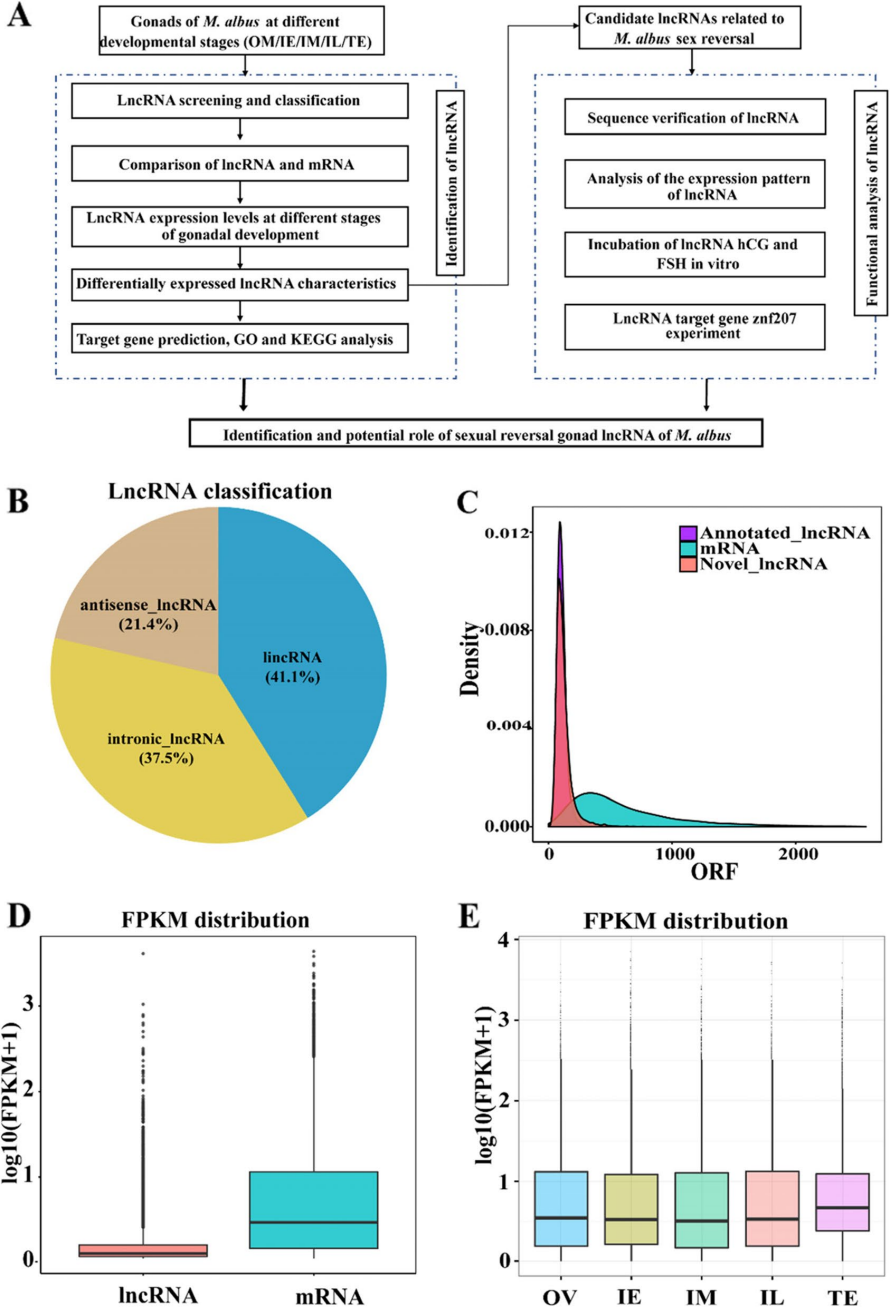
Global analysis of *M. albus* gonadal lncRNAs. **A** Experimental design diagram. **B** LncRNA type distribution map. **C** LncRNA and mRNA ORF length density map. **D** Expression levels of lncRNA and mRNA transcripts depicted as a violin plot. **E** Comparative analysis of sample expression levels. OV: ovary stage; IE: early intersexual stage; IM: middle intersexual stage; IL: late intersexual stage; TE: testis stage

In this study, a total of 1503 known lncRNAs and 11,243 new lncRNAs (Additional file 2: Fig. S1) were identified in libraries constructed from *M. albus* gonads. The whole experimental process is shown in Fig. 1A. After each step of screening, the final proportion of different types of lncRNAs (Fig. 1B), including lincRNAs (41.1%), antisense lncRNAs (21.4%), and intronic lncRNAs (37.5%), was determined. The ORF length and average FPKM of lncRNAs were smaller than those of mRNAs (Fig. 1C-D). By comparing the box plots of the quantitative results for different samples (Fig. 1E), the overall expression level in the male stage among different samples was found to be higher than that in the female/intersex stages (*P* < 0.05).

**Fig. 2:**
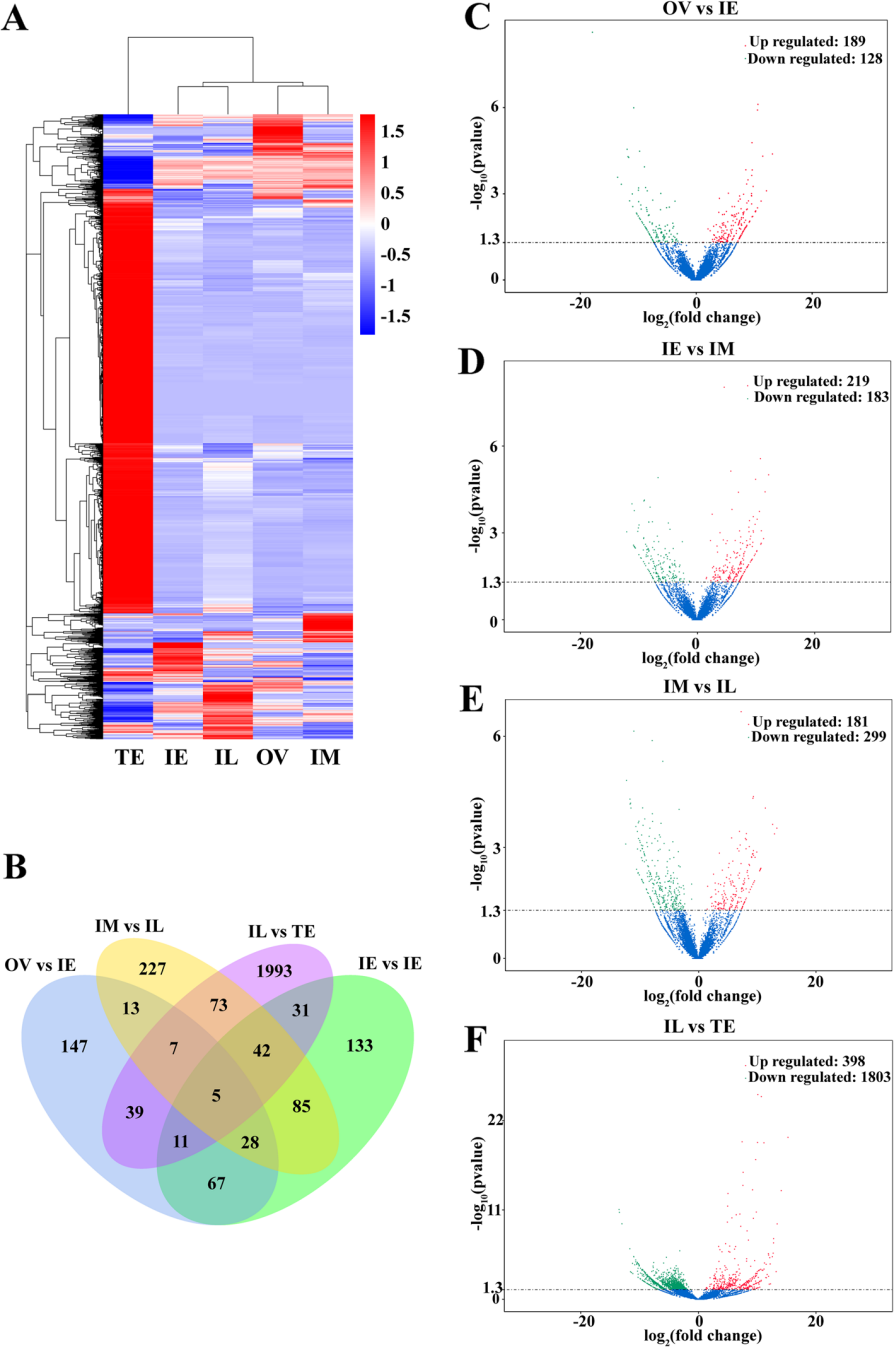
Differentially expressed lncRNAs (DE-lncRNAs) analyses. **A** Clustering of DE-lncRNAs. **B** Venn of DE-lncRNAs. **C-F** Volcano maps of DE-lncRNAs. OV: ovary stage; IE: early intersexual stage; IM: middle intersexual stage; IL: late intersexual stage; TE: testis stage

The DE-lncRNAs (*P* < 0.05) were counted according to the gonadal development of *M. albus* (Fig. 2, Additional file 3: Dataset S1). The aggregation of DE-lncRNAs at different gonadal stages indicated that ovarian and intersex expression clustered into one branch, and testis into one branch (Fig. 2A). Venn diagram analysis showed that there was a total of 2901 DE-lncRNAs, and 5 lncRNAs were differentially expressed throughout gonadal development (Fig. 2B). In OV vs. IE, 189 lncRNAs were up regulated and 128 lncRNAs were down regulated (Fig. 2C); in IE vs. IM, 219 lncRNAs were up regulated and 183 lncRNAs were down regulated (Fig. 2D); in IM vs. IL, 181 lncRNAs were up regulated and 299 lncRNAs were down regulated (Fig. 2E); and in IL vs. TE, 398 lncRNAs were up regulated and 1803 lncRNAs were down regulated (Fig. 2F). The results showed that the number of DE-lncRNAs gradually increased from the female to intersex to male stages, especially the number of DE-lncRNAs from IL to TE.

### GO and KEGG analyses of lncRNA target genes

In this study, we predicted the *trans*-target genes of lncRNAs, predicted the potential function of lncRNAs, and performed Gene Ontology (GO) cluster analysis (Table 1, Additional file 4: Fig. S2, Additional file 5: Dataset S2, Additional file 6: Dataset S3) and Kyoto Encyclopedia of Genes and Genomes (KEGG) enrichment analysis (Table 2, Additional file 7: Fig. S3, Additional file 8: Dataset S4, Additional file 6: Dataset S3). The significantly enriched (*q*-value< 0.05) GO terms were involved in regulation of cellular processes, regulation of biological processes and cellular components, and some *trans*-target genes of the lncRNAs were related to gonadal development, such as *foxo1*, *foxm1*, *smad3*, *foxr1*, *camk4* and *ar*. Genes that were significantly enriched in several KEGG pathways (*P* < 0.05) were identified as being involved in gonadal development, including *XLOC_059262* and its target *foxo1* in the insulin signalling pathway, *XLOC_091218* and its target *tgfb3* in the MAPK signalling pathway, and *XLOC_169543* and its target *camk4* in the calcium signalling pathway. Significantly, *XLOC_181771* and its target *smad3* and *XLOC_091218* and its target *tgfb3* were enriched in endocytosis pathways.

**Table 1:** DE-lncRNAs and their targets among the significantly enriched GO terms

Comparison	DE-lncRNA (number)	***Trans***-targets	GO terms	Term type
OV vs. IE	*XLOC_059262*(45)	*foxo1*	Regulation of biological process	BP
	*XLOC_138801*(29)	*foxm1*	Regulation of cellular process	BP
IE vs. IM	*XLOC_181771*(35)	*smad3*	Regulation of cellular process	BP
	*XLOC_138801*(29)	*foxm1*	Regulation of biological process	BP
			Biological regulation	BP
	*XLOC_064006*(2)	*foxr1*	Regulation of biological process	BP
			Biological regulation	BP
IM vs. IL	*XLOC_059262*(45)	*foxo1*	Regulation of biological process	BP
	*XLOC_181771*(35)	*smad3*	Regulation of biological process	BP
	*XLOC_138801*(29)	*foxm1*	Regulation of cellular process	BP
			Biological regulation	BP
	*XLOC_169543*(38)	*camk4*	Cellular process	BP
IL vs. TE	*XLOC_059262*(45)	*foxo1*	Transmembrane signaling	MF
			Receptor activity	MF
	*XLOC_181771*(35)	*smad3*	Regulation of cellular process	BP
			Biological regulation	BP
	*XLOC_138801*(29)	*foxm1*	Biological regulation	BP
	*XLOC_169543*(38)	*camk4*	Membrane	CC
	109,964,303 (3)	*ar*	Signaling receptor activity	MF
			Signal transducer activity	MF
			Receptor activity	MF
			Molecular transducer activity	MF
			Signal transduction	BP
			Single organism signaling	BP
			Signaling	BP
			Cell communication	BP
			Regulation of biological process	BP
			Cellular response to stimulus	BP
			Response to stimulus	BP

Note: lncRNA (number): the listed lncRNA Gene IDs are those with the smallest *P*-values, and numbers in parentheses are the numbers of lncRNAs; see Schedule Data 4 for details. *OV O*vary stage, *IE E*arly intersexual stage, *IM M*iddle intersexual stage, *IL L*ate intersexual stage, *TE T*estis stage. *BP B*iological process, *CC C*ellular component, *MF M*olecular function

**Table 2:** DE-lncRNAs and their targets among the significantly enriched KEGG pathways

Comparison	lncRNA (number)	***Trans***-targets	KEGG
OV vs. IE/ IE vs. IM/ IM vs. IL/ IL vs. TE	*XLOC_059262*(45)	*foxo1*	Insulin signaling pathway
	*XLOC_091218*(40)	*tgfb3*	MAPK signaling pathway
	*XLOC_169543*(38)	*camk4*	Calcium signaling pathway
OV vs. IE/ IL vs TE	*XLOC_181771*(35)	*smad3*	Endocytosis
	*XLOC_091218*(40)	*tgfb3*	Endocytosis

Note: lncRNA (number): the listed lncRNA Gene IDs are those with the smallest *P*-values, and numbers in parentheses are the numbers of lncRNAs; see Schedule Data 4 for details. *OV O*vary stage, *IE E*arly intersexual stage, *IM M*iddle intersexual stage, *IL L*ate intersexual stage, *TE T*estis stage

### Validation of DE-lncRNAs

**Fig. 3:**
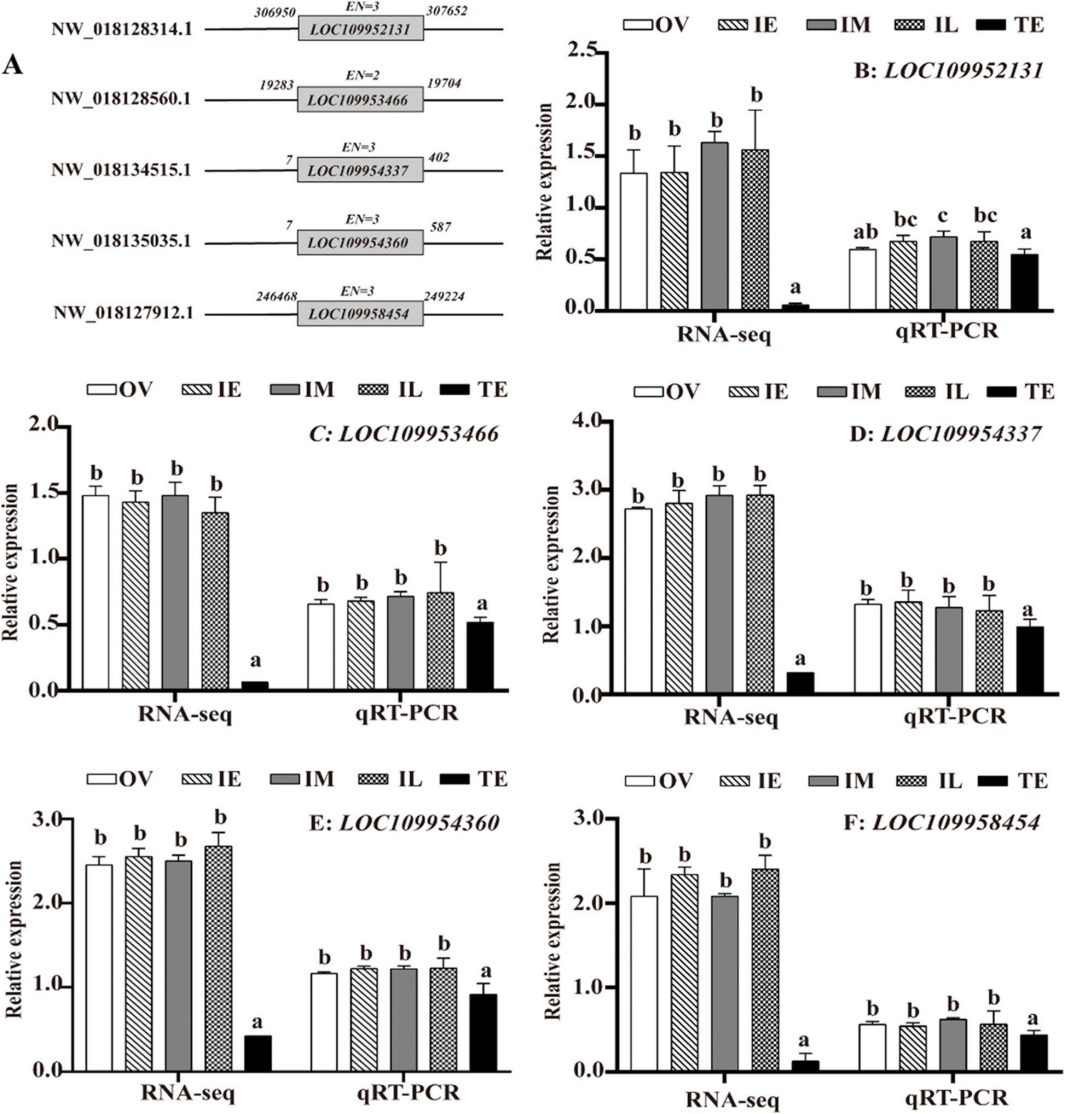
Verification of 5 DE-lncRNAs. **A** The location of lncRNAs on the *M. albus* chromosome. **B-F** Expression of 5 DE-lncRNAs in the gonads. qRT-PCR: real-time fluorescence quantification; RNA-seq: RNA sequencing. *EN*: exon number. OV: ovary stage; IE: early intersexual stage; IM: middle intersexual stage; IL: late intersexual stage; TE: testis stage. The results are presented as the means ± SEMs (*n =* 3). Means marked with different letters are significantly different (*P* < 0.05)

Through PCR technology, we successfully amplified 5 highly expressed DE-lncRNAs, namely, *LOC109952131* (450 bp), *LOC109953466* (320 bp), *LOC109954337* (239 bp), *LOC109954360* (424 bp) and *LOC109958454* (454 bp). In addition, we showed the location of the lncRNAs on the *M. albus* chromosome and their exon number (Fig. 3A).

To verify the authenticity of the RNA-seq data, we conducted qRT-PCR analysis to determine the expression levels of 5 randomly selected DE-lncRNAs in the gonads at the 5 developmental stages. The lncRNA expression trend obtained by qRT-PCR was consistent with the RNA-seq results, confirming that the RNA-seq data were reliable (Fig. 3 B-F). Five DE-lncRNAs were expressed at significantly lower levels in the male stage than in the female and intersex stages (*P* < 0.05). The significant difference in expression between the male and female/intersex stages further indicated that lncRNAs may regulate the gonadal development of *M. albus*.

### Expression of five DE-lncRNAs in the ovary after incubation with FSH and hCG

**Fig. 4:**
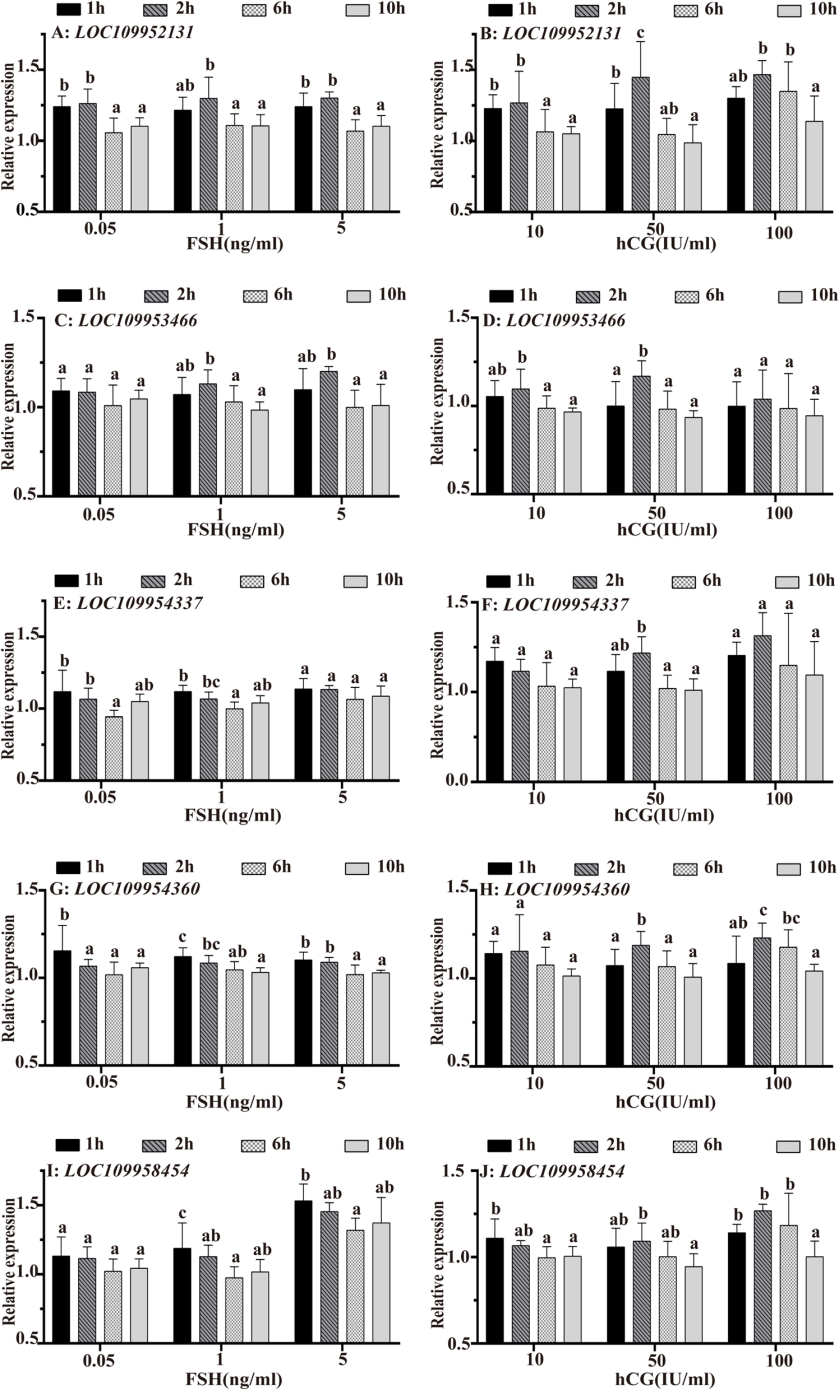
In vitro regulatory effect of hCG and FSH on the expression of DE-lncRNAs in the ovaries of *M. albus*. **A, B** *LOC109952131* expression patterns in ovaries after incubation with FSH and hCG. **C, D** *LOC109953466* expression pattern in ovaries after incubation with FSH and hCG. **E, F** *LOC109954337* expression pattern in ovaries after incubation with FSH and hCG. **G, H** *LOC109954360* expression pattern in ovaries after incubation with FSH and hCG. **I, J** *LOC109958454* expression pattern in ovaries after incubation with FSH and hCG. The results are presented as the means ± SEMs (*n =* 3). Means marked with different letters are significantly different (*P* < 0.05)

Incubation with FSH increased lncRNA expression in a time-dependent manner, and the overall trend was a decrease followed by an increase (Fig. 4E, G and I). Except for *LOC109952131* (Fig. 4A) and *LOC109953466* (Fig. 4C) incubated at 1 ng/mL and 5 ng/mL, all the samples showed the highest expression level at 1 h of incubation. Incubation with hCG increased lncRNA expression in a time-dependent manner, and the overall trend was an increase followed by a decrease (Fig. 4B, D and H). Except for *LOC109954337* (Fig. 4F) incubated at 10.0 IU/mL and *LOC109954454* (Fig. 4J) incubated at 10.0 IU/mL, all the samples showed the highest expression levels at 2 h of incubation.

### Identification of the *LOC109958454* target gene *znf207*

**Fig. 5:**
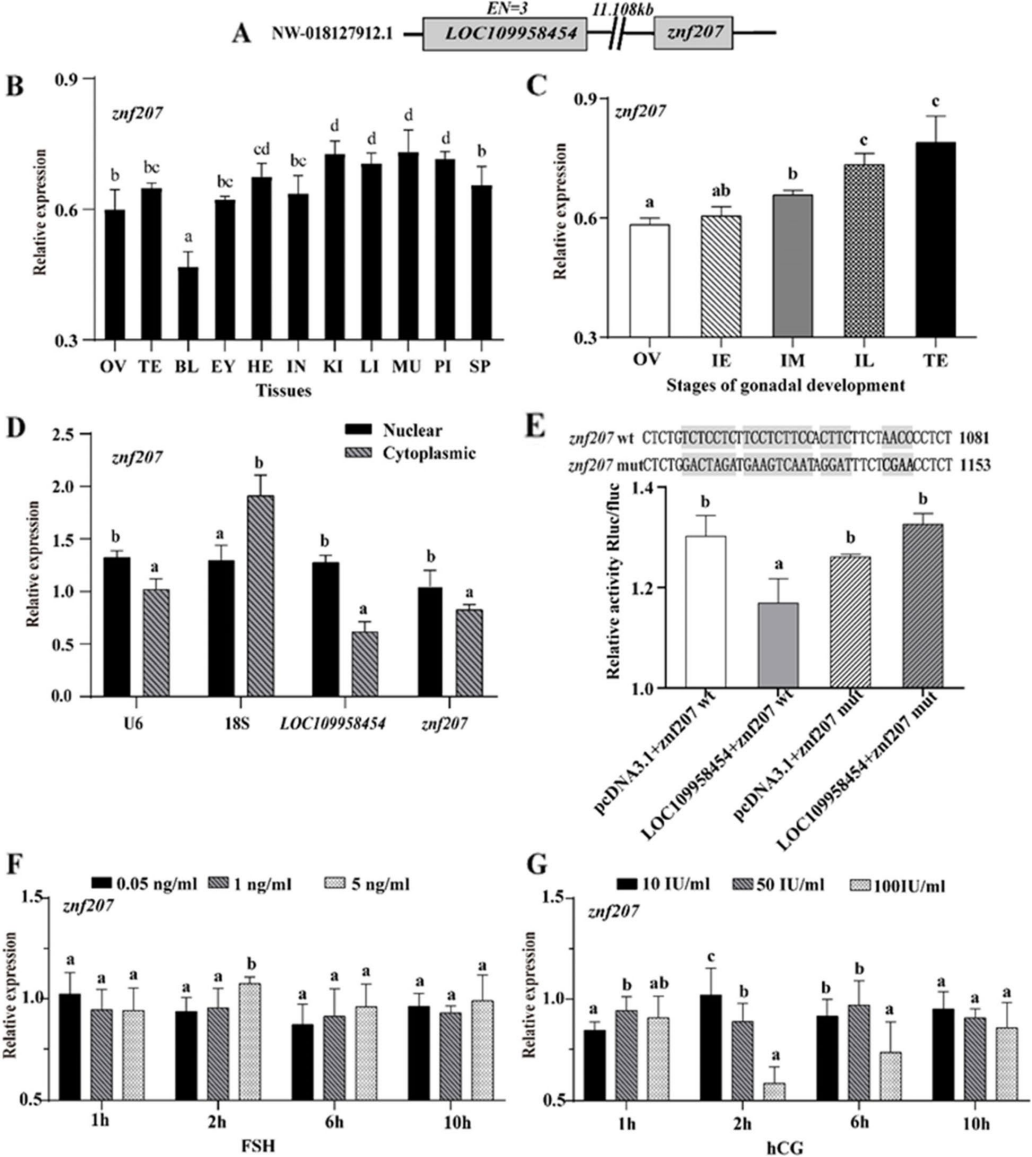
Expression level of the *LOC109958454 cis*-target gene *znf207.* **A** Positions of *LOC109958454* and *znf207* on the chromosome. **B, C** *Znf207* expression pattern analysis. **D** *LOC109958454* and *znf207* subcellular localization. **E** *LOC109958454* and *znf207* targeting verification*.* **F, G** In vitro regulatory effect of hCG and FSH on the expression of DE-lncRNAs in the gonads of *M. albus.* OV: ovary stage; IE: early intersexual stage; IM: middle intersexual stage; IL: late intersexual stage; TE: testis stage; PI: pituitary gland; EY: eye; HE: heart; LI: liver; KI: kidney; IN: intestines; SP: spleen; MU: muscle; Bl: blood. The results are presented as the means ± SEMs (*n =* 3). Means marked with different letters are significantly different (*P* < 0.05)

As shown in Fig. 5A, *LOC109958454* is 11.108 kb away from its colocalized mRNA *znf207*. The results of tissue distribution experiments showed that *znf207* was widely distributed in gonadal tissues (ovary and testis) and nongonadal tissues (eyes, pituitary gland, heart, etc.) of *M. albus* (Fig. 5B). In contrast to *LOC109958454*, the expression of *znf207* continued to increase, and the expression in the male stage was higher than that in the female and intersex stages during the gonadal development process (Fig. 5C). The biological relationship of lncRNAs is often related to their subcellular location. The results showed that *LOC109958454* and *znf207* were specifically expressed in the nucleus (Fig. 5D). The results of the dual-luciferase assay showed that *LOC109958454* could inhibit the expression of *znf207,* and this inhibition disappeared or was weakened when the binding site was mutated (Fig. 5E). The expression of *znf207* at each time point after incubation with FSH and hCG divided by that in the control group (0 h) was mostly less than 1. FSH and hCG inhibited the expression of *znf207* (Fig. 5F-G). In short, *znf207* is the target gene of *LOC109958454*, and its expression level is affected by hormones.

## Discussion

### Function of lncRNAs in gonadal development

Transcriptome sequencing of Meishan pig and Yorkshire ovarian tissues revealed 3827 lncRNAs and 192 DE-lncRNAs [25]. In Duroc pigs ovaries on days 0, 2, and 4 of follicular development, 140 lncRNAs were found to be differentially expressed in pairwise comparisons [26]. There were 115 DE-lncRNAs between the goat ovarian follicular phase and luteal phase [27]. A total of 1118 DE-lncRNAs were found based on the comparison between the testes of 3 month old and 9 month old sheep [28]. In our research, the number of DE-lncRNAs corresponding to OV vs. IE, IE vs. IM, IM vs. IL and IL vs. TE was 317, 402, 408, and 2201, respectively. Analysis of the results of lncRNA identification in different species and the differential expression at different developmental stages suggested that lncRNAs also play roles in gonadal development in *M. albus*.

LncRNAs have a wide range of functions in animal reproduction and development, including in germ cell growth, meiosis, gametogenesis, sex hormone response, and sex determination [29–31]. A recent study provided the first evidence that the lncRNA *H19* is associated with polycystic ovary syndrome (PCOS) in women and that increased levels of lncRNA *H19* are a risk factor for PCOS [32]. The expression of lncRNAs (such as *XR_001917388.1*) in goat ovaries during the follicular phase is higher than that in the luteal phase and may regulate oogenesis and oocyte maturation [27]. The lncRNA *TCONS_00030774* is located approximately 500 bp downstream of the estrogen receptor 1 (*ESR1*), which indicates that it plays a role in porcine ovarian development [25]. The lncRNAs *1700108J01Rik* and *1700101O22Rik*, both of which are mouse testis specific lncRNAs, are specifically expressed in the testicular germ cells of premeiosis and round spermatids, which coincides with the reactivation of transcription during spermatogenesis [16]. The lncRNA *NONMMUT074098.2* (*Lnc10*) can cause abnormal spermatogenesis by promoting germ cell apoptosis. After *Lnc10* was knocked down, the morphology of the testis changes, and the average quality decreases [33]. In addition, lncRNAs can regulate the gonadal development of *Mauremys mutica* [14], *P. sinensis* [34] and *Scylla paramamosain* [35] and affect sex differentiation. In our study, the *cis*-target genes of the lncRNAs included a large number of genes involved in gonadal development, including *igf1* [36], *sox9* [37], *gdf9* [38], and *smad2* [24] (Additional file 9: Dataset S5). Therefore, lncRNAs may play roles in the gonadal development process in *M. albus*, and the specific mechanism needs to be further studied.

### DE-lncRNA target genes and their GO and KEGG analyses

A total of 12,746 lncRNAs were identified from the gonads of *M. albus* by RNA-seq, and 2901of the lncRNAs were differentially expressed. In this study, some of the predicted *trans*-target genes were related to gonadal development, such as *foxo1*, *foxm1, smad3*, *foxr1*, *camk4*, *ar* and *tgfb3* (Table 1 and Table 2). The *XLOC_059262* target gene *foxo1*, *XLOC_138801* target gene *foxm1* and *XLOC_064006* target gene *foxr1* are all fox genes, which play important roles in the sex determination and gonadal development of bony fish [39]. The target gene of *XLOC_181771*, *smad3*, is involved in endocytosis and is also a member of the TGF-β family. That the gene *smad3* may be the main signal transduction molecule that, via activin, stimulates the expression of FSHβ in goldfish [40]. The target gene *camk4* of *XLOC_169543* in the calcium signalling pathway is expressed at high levels. In mice lacking *camk4*, spermatogenesis and basic nucleoprotein exchange are impaired, leading to male sterility [41]. Similarly, female fertility in *camk4* deficient muscle groups is significantly reduced due to follicular development and ovulation damage [42]. *ar* is an important receptor in the supporting cells of the testis. It plays an important role in maintaining the number of spermatogonia and the integrity of the blood testis barrier, in the completion of meiosis, in the adhesion of sperm cells and in the fertilization of sperm and other spermatogenesis processes [43, 44]. In this study, *ar* was enriched in only IL vs. TE, indicating that it plays an important role in the development of the testis during the sex reversal process of *M. albus*. Mutation of the *XLOC_003298* target gene *tgfb3* may be related to male sterility [45]. In addition, *tgfb3* has a key role related to meiotic arrest and the development of oocytes [46, 47].

The predicted target genes of the DE-lncRNAs were analysed by GO and KEGG cluster analyses. In our research, the insulin signalling pathway, MAPK signalling pathway, and calcium signalling pathway were found to be enriched. The lncRNA GAS5 may regulate the expression of PARP1 by recruiting the transcription factor E2F4 to its promoter, affecting the activity of the MAPK signalling pathway, promoting apoptosis and causing G0/G1 arrest in ovarian cancer cells [48]. The results in zebrafish (*Danio rerio*) show that Corticotropin-releasing hormone (CRH) and its receptor inhibit estrogen production and synthesis, and the inhibition of CRHα is partly mediated through the p38 MAPK signalling pathway [49]. Injection of IRS (an insulin signaling pathway component) dsRNA into adult female oriental fruit fly significantly reduced IRS transcript levels, thereby inhibiting ovarian development, and the average size of the ovaries was reduced by 33% compared to controls [50]. A calcium signaling pathway, initiated by internal stores, is required for acute LH-induced steroidogenesis [51]. And calcium is also an important component of the signal transduction pathway, which is an important regulatory component of meiosis during oogenesis in mouse [52]. In our previous study, insulin, MAPK and calcium signalling pathway were involved in the process of *M. albus* cell apoptosis [53]. In this study, these pathways were also enriched in the whole process of sexual reversal of *M. albus*, which suggested that it may be involved in the whole process of gonadal development, especially in the process of ovarian development and apoptosis. Receptor-mediated endocytosis uptake of multiple ligands, such as hormones, growth factors, and transport molecules, is required for gonadal development [54]. In teleost fish, vitellogenin (Vtg) is released into the blood circulation through Vtg receptor-mediated endocytosis, and combines with oocytes to form yolk granules, thereby participating in fish ovary growth [55]. In our previous report, endocytosis was associated with oocyte apoptosis during the female-to-male sex transition of *M. albus* [53]. In this study, endocytosis was enriched during the gonad development from OV to IE and from IL to TE, indicating that endocytosis may be involved in the process of oocyte development and sexual reversal of *M. albus*. The specific mechanism remains to be further explored.

### Effect of FSH and hCG on lncRNAs

FSH and hCG levels can affect gonadal development [56, 57]. Studies have found that FSH can promote the growth of *M. albus* oocytes, playing an important role in the transition from primary follicle growth to secondary follicle growth, which is an important step in oocyte maturation [58]. hCG promotes gonad maturation in *Trachinotus blochii* [59] and *Anguilla japonica* [60]. Interestingly, the expression levels of lncRNAs can affect the levels of hormones (such as FSH) and are regulated by hormones (such as hCG). For example, after interfering with the expression of the lncRNA *TCONS_00066406*, the expression level of FSH is reduced, affecting female reproduction [61, 62]. Moreover, the lncRNA *HAS2-as1* is an hCG target that promotes the expression of HAS2 and may play a role in regulating cumulus expansion and migration [63]. In our previous study, FSH and hCG were used to promote the expression of the lncRNA *LOC109960696 trans*-target gene *Smad2* in *M. albus* ovarian tissue in a time- and dose-dependent manner, respectively [24]. We successfully amplified 5 highly expressed DE-lncRNAs (*LOC109952131*, *LOC109953466*, *LOC109954337, LOC109954360* and *LOC109958454*). The results showed that the 5 lncRNAs were expressed at higher levels in the ovary and intersexual stage than in the testis. Incubation with FSH and hCG increased the expression of the 5 lncRNAs in a time-dependent manner. In summary, it was shown that the expression of lncRNAs is regulated by the levels of hormones, which in turn affects gonadal development in organisms.

### Potential effect of the *LOC109958454 cis*-target gene *znf207* on gonadal development

Many scholars have also explored the interactions between target genes and lncRNAs. In *C. livia*, RNA-seq analysis showed that *FOXK2* was a target gene of the lncRNA *MSTRG.7894.4* involved in the regulation of the oestrogen receptor, and qRT-PCR experiments showed that the mRNA expression level of the *ERα* gene in the high egg production performance group was significantly higher than that in the low egg production performance group [17, 64]. In *S. paramamosain*, the expression level and complementary sequence of lncRNA-*ncr2* were found to be higher in the ovary than in the testis, and the expression levels of its target genes *Sp-jcb2* and *Sp-jcb3* in the ovary were significantly lower than those in the testis [35]. In *Fenneropenaeus merguiensis*, the expression of *lncPV13* was inhibited and the expression of vitellogenin (Vg) was significantly decreased in the ovary on day 7 after injection of lncPV13-specific double-stranded RNA (*dslncPV13*). In contrast, the expression of gonad-inhibiting hormone (GIH) was significantly increased in *lncPV13*-knockout shrimp [65]. We found that the *cis*-target gene *znf207* was located 11.108 kb downstream of *LOC109958454*. This gene was widely expressed in various tissues of *M. albus*, and its expression in the testis was higher than that in the intersex stages and ovaries.

*Znf207* belongs to the zinc finger protein family, members of which are involved in a variety of biological processes, including chromatin remodelling, transcription activation/inhibition, DNA repair, apoptosis regulation, protein folding and assembly, stress response, and cell proliferation and differentiation [66, 67]. Nuclear lncRNAs are involved in a variety of biological processes, including chromatin organization, transcription and posttranscriptional gene expression, and serve as structural scaffolds for the nuclear domain [68]. Depletion of *znf207* leads to an increase in the formation of RNA-DNA hybrids (R-loops), which leads to DNA damage and p53 activation in cells, which in turn leads to cell apoptosis or senescence [69]. Loss of *znf207* gene expression is beneficial for the treatment of *tp53* mutant ovarian cancer [70]. In our study, *LOC109958454* and *znf207* were found to be expressed mainly in the nucleus and to have a targeting relationship; *LOC109958454* inhibits the expression of *znf207.* In addition, FSH and hCG inhibited the expression of *znf207*. The lncRNA and hormones could regulate the expression of *znf207*; the specific mechanism remains to be further explored.

## Conclusion

To the best of our knowledge, our study is the first systematic report of lncRNA sequencing for the five stages of gonadal development during sexual reversal of *M. albus*, and many candidate lncRNAs related to gonadal development were discovered. We showed that 5 lncRNAs had higher levels in the female and intersex stages and that their expression levels were affected by the hormones FSH and hCG. And *LOC109958454* and *znf207* were expressed mainly in the nucleus rather than the cytoplasm, verifying their targeting relationship, and found that the expression of *znf207* was suppressed by hormones. The above experiments showed that many lncRNAs are involved in the gonadal development of *M. albus* and may function through target genes. This study provides a new theoretical basis for the study of gonadal development in teleost fish and other animals.

## Methods

### Sample collection and preparation

Wild *M. albus* (*n =* 300) were purchased from a local market in Chengdu, Sichuan. These fish were kept under the natural temperature and photoperiod in the laboratory. All procedures and investigations used for animal research were subject to approval and performed in accordance with the guidelines of the ethics committee (Approval No.20190031).

Fish were anaesthetized with 0.02% tricaine buffer (80 μg/L) (Sigma, LA, USA) for 10 min after a 24 h fast, and the tissues, including half of the gonads, pituitary gland, eye, heart, kidney, intestines, spleen, muscle and blood, were collected and immediately stored in liquid nitrogen at − 80 °C. The other half of the fresh gonads were immediately fixed in Bouin’s solution for 24 h and then stored in 75% ethanol until paraffin embedding.

**Fig. 6:**
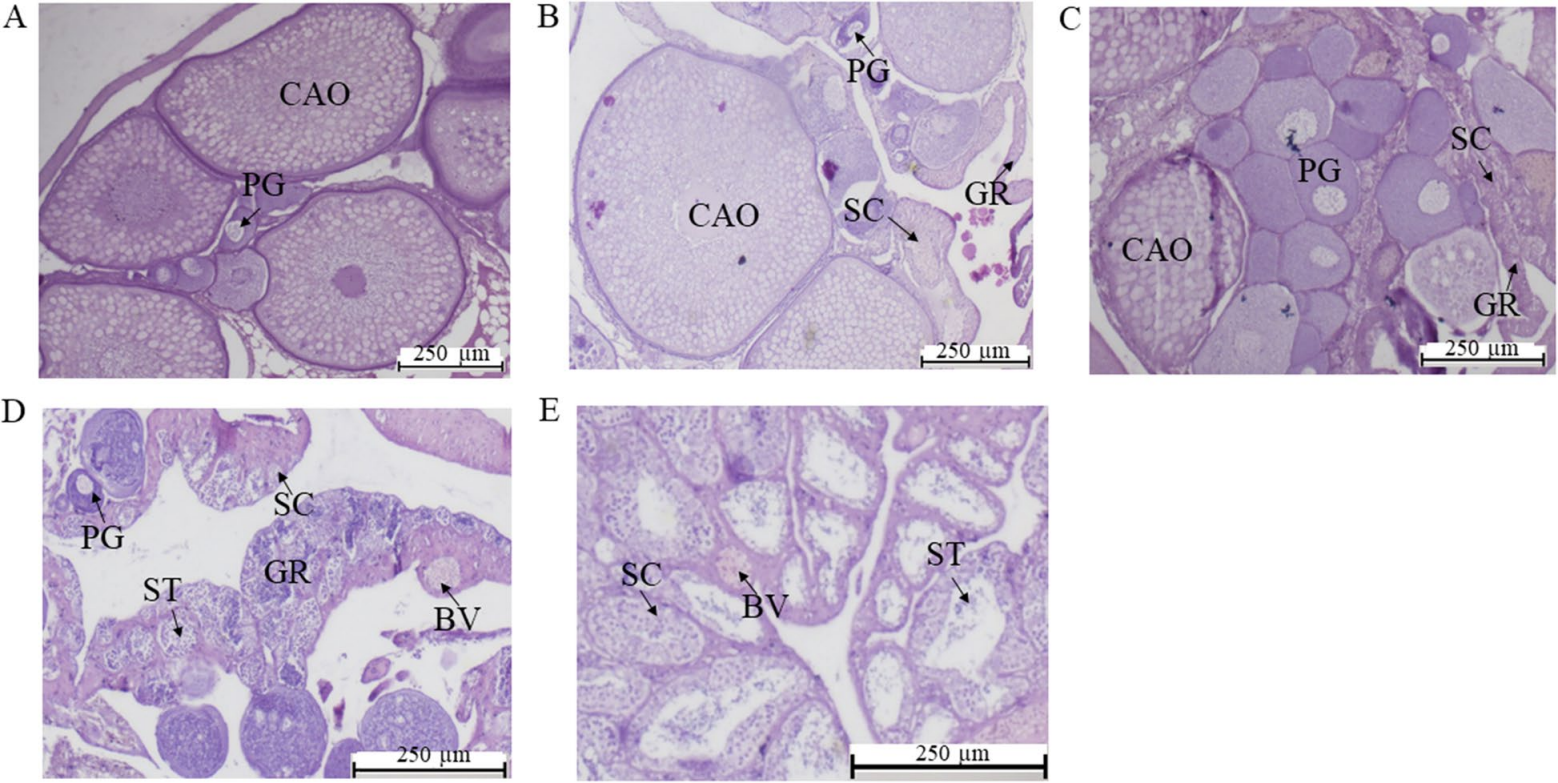
Results of identification of the gonad development stages of *M. albus*. **A**: Ovary stage (OV), **B**: early intersexual gonad (IE), **C**: middle intersexual gonad (IM), **D**: late intersexual gonad (IL), **E**: testis stage (TE). CAO: cortical alveolar oocyte, PG: primary growth stage, GR: gonadal ridge, SC: spermatocyte, ST: spermatid, BV: blood vessel

Sections were serially cut at a thickness of 5 μm using a slicer (Leica, Nussloch, Germany) and stained with haematoxylin/eosin [24]. Through gonadal histological sectioning, gonadal samples from different stages of the *M. albus* sex reversal process were verified. With reference to the previuos reports [24, 45, 71], according to the proportion of male and female germ cell development, the transition from female to male sex was classified into five phases: ovary in mature stage (OV) (Fig. 6A), early intersexual stage gonad (IE) (Fig. 6B), middle intersexual stage gonad (IM) (Fig. 6C), late intersexual stage gonad (IL) (Fig. 6D), and testis (TE) (Fig. 6E).

### Sequencing method and quality control

We used *M. albus* gonad samples for RNA-seq at the 5 developmental stages, with 3 biological replicates in each stage, and for identification and analysis of lncRNAs. Raw data in FASTQ format were first processed through in-house Perl scripts. In this step, clean data were obtained by removing reads containing adapters, reads containing poly-N sequences and low-quality reads from the raw data. At the same time, the Q20, Q30 and GC contents of the clean data were calculated. All subsequent analyses were based on clean data of high quality.

### LncRNA identification

Cuffmerge was used to merge the transcripts spliced from each sample, and the transcripts with uncertain chain directions were removed to obtain the complete transcriptome information from this sequencing data [72]. Finally, five basic principles were used to filter the data: (1) transcripts with exon number ≥ 2; (2) transcripts with transcript length > 200 bp; (3) screening of transcripts with known annotations; (4) calculation of the expression of each transcript by Cuffquant, selecting transcripts with fragments per kilobase of transcript per million mapped reads (FPKM) value ≥0.5; and (5) use of Coding Potential Calculator (CPC) [73] to filter transcripts with coding potential.

### Differential expression of lncRNAs

After screening, String Tie-eB [74] software was used to quantitatively analyse the transcripts, including lncRNAs, and obtain the FPKM values of the transcripts of each sample. With regard to statistical significance, lncRNAs were analysed as a whole. The DE-lncRNAs were statistically analysed using the edgeR program with default parameters: *p*-value < 0.05 and |log_2_ (fold change)| > 1.

### Target gene prediction, GO and KEGG enrichment analyses

For position-related target gene analysis, the *cis*-target genes were predicted based on the positional relationship between lncRNAs and mRNAs, and the screening range was within 100 kb. Target gene prediction was based on the expression correlation between lncRNAs and mRNAs, and the screening condition was Pearson’s correlation coefficient > 0.95. It should be noted that the mRNA data came from our laboratory, and RNA-seq was performed using the same batch of samples as those used for lncRNA analysis. GO enrichment analysis of DE-lncRNA target genes was performed by the GOseq R software package [75], and GO entries with *q*-value < 0.05 were considered to be significantly enriched. KEGG is a high-throughput experimental database generated from a large-scale molecular data set by using a bioinformatics database resource for biological systems. A pathway enriched with *P* < 0.05 was defined as being significantly enriched by the associated differentially expressed gene. We used KOBAS software [76] to detect the enrichment of KEGG pathways by lncRNA target genes.

### Cloning of 5 full length DE-lncRNAs

Using gonadal cDNAs as templates and the primer sequences shown in Additional file 10: Table S2, the initial fragments of the full length DE-lncRNAs were amplified by nested PCR. The final volume of the PCR mixture was 20 μL, which contained 2 μL of gonadal cDNA, 1 μL of each of the upstream and downstream primers (10 μM), 6 μL of water, and 10 μL of 2 × PCR Mix (Vazyme, Nanjing, China). The PCR program included 95 °C for 3 min, 36 cycles (95 °C for 0.5 min, TMC for 0.5 min, 72 °C for 1.5 min), at 72 °C for 10 min and storage at 4 °C until use. The gel recovery product was cloned into the pMD-19 T vector (Vazyme, Nanjing, China) and sequenced using universal primers.

### Expression patterns of the DE-lncRNAs determined by qRT-PCR

cDNA was obtained from gonadal and other tissues (pituitary gland, eye, heart, kidney, intestines, spleen, muscle, blood) at the five developmental stages. qRT-PCR analysis (by the double internal reference method) was used to determine the expression levels of lncRNAs in the gonads at the different developmental stages. The genes *ef1α* and *rpl17* (the GenBank accession numbers are kc011266 and kc011267, respectively) were identified as the most stable genes in *M. albus* at different stages of gonadal development and in different tissues [77] and were therefore used as internal controls. The primer sequences are shown in Additional file 11: Table S3. The CFX system (Bio–Rad, Chicago, USA) was used for qRT-PCR. The PCR mixture contained 1 μL of cDNA template, 0.4 μM upstream and downstream primers, and 10 μL of 2× SYBR Green MasterMix (TaKaRa Bio, Dalian, China). PCR was carried out at 95 °C for 2 min, followed by 40 cycles of 95 °C for 0.5 s and 57-61 °C for 0.5 min and then by signal collection. The melting curve was generated as follows: 95 °C for 5 s, followed by heating from 65 °C-95 °C, increasing the temperature by 5 °C every 5 s, and then by signal collection. The amplification curve showed the expression levels and melting curve analysis confirmed the specificity of qRT-PCR amplification; CFX Manager software was used to analyse gene expression. Calculation of the copy numbers of the lncRNA and internal reference genes in CFX Manager was based on DNA fragments, ranging from 10^2^ to 10^9^ copies [38]. The transcriptional expression level of the DE-lncRNA was calculated as follows:  

$$C_{\ln cRNA}/\sqrt{Cef1\alpha\ast Crpl17}$$

The expression levels of the lncRNA target genes were determined by the same method.

### Expression patterns of 5 lncRNAs and *znf207* after incubating ovaries with hCG and FSH in vitro

The expression patterns of 5 DE-lncRNAs (*LOC109952131*, *LOC109953466*, *LOC109954337, LOC109954360* and *LOC109958454*) and the target gene *znf207* of *LOC109958454* were analysed after incubating ovaries with human chorionic gonadotropin (hCG) and follicle-stimulating hormone (FSH) in vitro. M199 medium (Sigma, Shanghai, China) was applied on ice, and *M. albus* ovaries were washed and dissected. Ovarian tissue (50-100 mg) was placed in a 24-well petri dish, and 1 mL of L15 containing penicillin (0.1 μg/mL, Gibco, Massachusetts, USA) and streptomycin (0.1 mg/mL, Gibco, Massachusetts, USA) was added to the basal medium; tissue culture was performed at 28 °C in a humidified incubator. Stock solutions of hCG and FSH (Sigma, Shanghai, China) were prepared in saline solution at 10^3^ times higher than the final concentration. After pre-incubation in normal saline for 2 h, FSH (0.05, 1.0, or 5.0 ng/ml), hCG (10, 50, or 100 IU/ml) or normal saline (control group) were incubated for 1, 2, 4, or 10 h with 3 replicates per treatment. After incubation, total RNA from ovarian tissue was extracted with TRIzol reagent (Invitrogen, Chicago, USA), cDNA was synthesized by reverse transcription, and qRT-PCR was performed to analyse the expression of lncRNAs. The results of the experimental group were compared to those of the control group as the final mapping data. Please refer to section 2.7 for the method.

### Subcellular localization analysis of *LOC109958454* and *znf207*

The ovarian tissue samples of *M. albus* were cleaned and dissected and then placed in cold Leibovitz’s L-15 medium (Gibco, Massachusetts, USA). Tweezers were used to continuously peel off the oocytes and connective tissue. After mixing the oocytes, the samples were divided into 5 groups, taking care to not break the cells during this process. Resuspend the cell pellet in precooled PBS (Gibco, Massachusetts, USA), and then, prechilled HLB (Foregene, Chengdu, China) was added at least 5 times the volume of the cell pellet, followed by the addition of 100 U of RNase inhibitor (G-Clone, Beijing, China). The mixture was vortexed for 10 s and centrifuged at 1000×g and 4 °C for 5 min. The supernatant (cytoplasm) was carefully aspirated into a new 1.5 mL EP tube, and the pellet (nucleus) was washed with 1 mL of HLB. An RNAprep Pure Micro Kit (Tiangen, Beijing, China) was used to extract cytoplasmic and nuclear RNA, which was then reverse transcribed to cDNA; *U6* and *18S* were used as internal reference genes to perform qRT-PCR experiments on the lncRNAs. Please refer to section 2.7 for the method.

### Plasmid construction and dual-luciferase assay

The full length *LOC109958454*, *znf207* and *znf207 mut* cDNAs were amplified from *M. albus* and cloned into the PSE5390 vector (Sangon Biotech, Shanghai, China) to obtain PSE5390-*LOC109958454*, PSE5390-*znf207* and PSE5390-*znf207 mut*, respectively. Then, 293 T cells (Sangon Biotech, Shanghai, China) were inoculated into a 24 well plate at 30-50% confluence, with 3 replicate wells for each group. Cell transfection was performed using Lipo2000 (Invitrogen, Chicago, USA). Then, 200 μL of diluted 1× PLB was added to each well for cell lysis, and 20 μL of LAR II was added to detect firefly luciferase activity with a microplate reader (Tecan, Shanghai, China). After adding 20 μL of Stop&Glo Reagent, the microplate reader was used to detect Renilla luciferase activity. The Renilla luciferase activity values were divided by the firefly luciferase activity values, and the average values were plotted.

### Statistical analysis

All the data are expressed as the mean ± S.E.M. The data were subjected to single-factor analysis of variance, followed by Tukey’s multiple comparison test, with SPSS 20.0 software (SPSS, Inc., Chicago, IL, USA). *P* < 0.05 was considered to indicate statistical significance.

## Supplementary Information

**Additional file 1: Table S1.** Quality inspection of transcriptome data.

**Additional file 2: Fig. S1.** Identification results of new lncRNA.

**Additional file 3: Dataset S1.** DE-lncRNA annotation.

**Additional file 4: Fig. S2.** GO enrichment analysis of DE-lncRNA *trans*-target genes.

**Additional file 5: Dataset S2.** GO terms with significant differences among different developmental stages.

**Additional file 6: Dataset S3.** DE-lncRNAs and their targets in the significantly enriched GO terms and KEGG pathways.

**Additional file 7: Fig. S3.** KEGG enrichment analysis of DE-lncRNA *trans*-target genes.

**Additional file 8: Dataset S4.** KEGG pathways with significant differences among different developmental stages.

**Additional file 9: Dataset S5.** DE-lncRNAs and their *cis*-target genes.

**Additional file 10: Table S2.** Primers used for cloning.

**Additional file 11: Table S3.** Primers used for qRT–PCR and incubation.

## Data Availability

The datasets generated and analysed during the current study are available in the Sequence Read Archive of National Center for Biotechnology Information database with accession number PRJNA798587 (https://dataview.ncbi.nlm.nih.gov/object/PRJNA798587). The datasets analysed during this study are included in this published article and its supplementary information files. Please contact Zhi He (zhihe@sicau.edu.cn) if someone wants to request the data from this study.

## Abbreviations

lncRNAs: Long noncoding RNAs
DE-lncRNAs: Differentially expressed lncRNAs
FSH: Follicle stimulating hormone
hCG: Human chorionic gonadotropin
qRT-PCR: Quantitative real-time PCR
OV: Ovary stage
IE: Early intersexual stage
IM: Middle intersexual stage
IL: Late intersexual stage
TE: Testis stage
GO: GENE Ontology
BP: Biological process
CC: Cellular component
MF: Molecular function
KEGG: Kyoto Encyclopedia of Genes and Genomes
EN: Exon number
PI: Pituitary gland
EY: Eye
HE: Heart
LI: Liver
KI: Kidney
IN: Intestines
SP: Spleen
MU: Muscle
Bl: Blood
PCOS: Polycystic ovary syndrome
ESR1: The estrogen receptor 1
LH: Luteinizing hormone
